# A rare case of pediatric inguinal hernia containing bilateral ovaries diagnosed intraoperatively

**DOI:** 10.1093/jscr/rjaf1010

**Published:** 2025-12-23

**Authors:** Ryoma Sakamoto, Satoshi Matsuda, Ryohei Uchimura, Hiroshi Kusanagi

**Affiliations:** Department of Gastrointestinal Surgery, Kameda Medical Center, 929, Higashimachi, Kamogawa, Chiba 2960041, Japan; Department of Gastrointestinal Surgery, Kameda Medical Center, 929, Higashimachi, Kamogawa, Chiba 2960041, Japan; Department of Pediatric Surgery, Kameda Medical Center, 929, Higashimashi, Kamogawa, Chiba 2960041, Japan; Department of Gastrointestinal Surgery, Kameda Medical Center, 929, Higashimachi, Kamogawa, Chiba 2960041, Japan; Department of Gastrointestinal Surgery, Kameda Medical Center, 929, Higashimachi, Kamogawa, Chiba 2960041, Japan

**Keywords:** inguinal hernia, ovary, preoperative diagnosis

## Abstract

Ovarian herniation is commonly observed in female infants with inguinal hernia; however, inguinal hernias involving both ovaries are extremely rare. A 6-year-old female with a history of low birth weight presented to our department with a left inguinal bulge. Ultrasonography revealed a highly echogenic mass in the hernia sac, raising the suspicion of herniation of the ipsilateral ovary. We performed a semi-urgent hernia repair. During the procedure, both ovaries were identified within a single sac. Few reports have described inguinal hernias involving both ovaries. Furthermore, previous cases were associated with prolapse of other organs, and to the best of our knowledge, we are the first to report a case of an inguinal hernia involving only both ovaries. Herniation of both ovaries is challenging to diagnose preoperatively, representing a potential pitfall of hernial repair in female infants.

## Introduction

Inguinal hernia is the most common anomaly in pediatric surgery, and inguinal hernias containing ovarian tissue account for 15%–20% of cases in female infants [1]. However, in most cases, the hernial sac contains only the ipsilateral ovary, and inguinal hernias involving both ovaries are extremely rare. Here, we report the case of a 6-month-old female with an inguinal hernia containing both ovaries, along with a review of the literature.

## Case report

**Figure 1:**
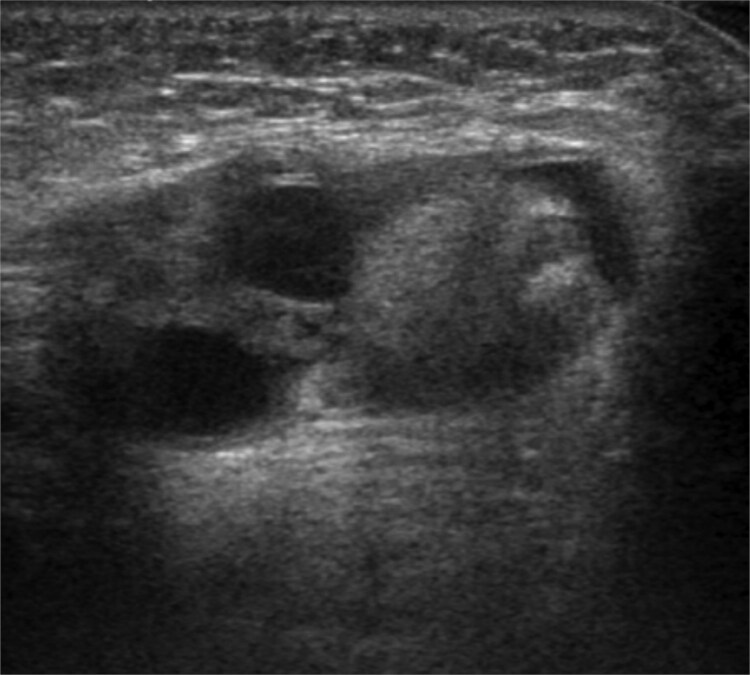
Ultrasonography revealed a 12 mm-sized circular highly echogenic mass and 7- and 8-mm-sized cystic masses within the hernia sac. Ipsilateral ovarian herniation was suspected.

**Figure 2:**
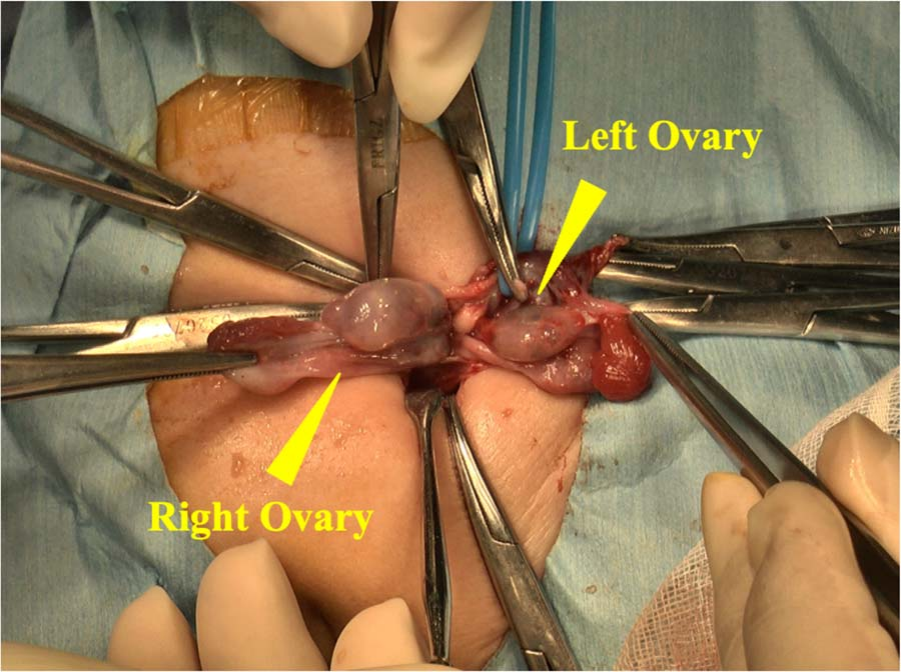
The hernia sac was opened during the procedure, revealing a sliding left ovary and a freely prolapsed right ovary.

A 6-month-old female with low birth weight (birth weight: 436 g, gestation period: 28 weeks and 6 days) and a history of neonatal intensive care unit admission visited our department due to a left inguinal bulge. Her medical history included premature ductus arteriosus, for which she underwent ligation surgery 5 days after birth. She also required ventilatory support and administration of sildenafil citrate after birth due to persistent neonatal pulmonary hypertension. At the time of presentation to our department, the patient required home oxygen therapy (0.5 L/min). Abdominal ultrasonography revealed a hernia sac in the left inguinal region. A single circular, highly echogenic mass was observed within the sac, raising suspicion of herniation of the ipsilateral ovary (Fig. 1). Considering the risk of torsion and necrosis of the affected ovary, semi-urgent hernia repair (Potts’ procedure) was performed 6 days after the initial presentation. During the procedure, we identified two ovaries in a single hernia sac. One ovary, the left, had prolapsed into the hernia sac due to sliding, while the other was the right ovary (Fig. 2). The right ovary exhibited no adhesions to the surrounding tissue and was manually repositioned in the abdominal cavity. The left ovary was returned to the abdominal cavity by applying a purse-string suture to the hernia sac at the peripheral margin of the ovarian attachment. The postoperative course was uneventful, and the patient was discharged 7 days following surgery. No complications occurred. At 1-year follow-up, the patient remained recurrence-free.

## Discussion

Prolapse of the ovary into the hernia sac is typically observed in 15%–20% of all cases of inguinal hernia in female infants [1]. Hirabayashi *et al.* suggested that emergency surgery for inguinal hernia with ovarian content in female infants is not always necessary, as the ovary spontaneously returns to the abdominal cavity in many cases [2]. Generally, prompt surgical intervention is recommended due to the risk of torsion and necrosis of the affected ovary [3, 4]. At our institution, we routinely perform semi-urgent surgery for inguinal hernias with ovarian contents. Unilateral inguinal hernias involving both ovaries are extremely rare, with only seven cases reported to date [5–11]. Six of these cases were accompanied by uterine prolapse [5–10], and in the remaining case [11], the left bladder ear and hemiuterus were noted within the hernia sac. To the best of our knowledge, inguinal hernias involving exclusively the bilateral ovaries have not been described previously, making this the first documented case. Okada *et al.* hypothesized that uterine and ovarian prolapse result from a combination of factors, including weak fixation of the uterus and ovaries, traction exerted by a previously prolapsed ipsilateral ovary, and elevated intra-abdominal pressure associated with crying [5]. In our case, following herniation of the uterus and ovaries, only the uterus spontaneously returned to the abdominal cavity. In the future, laparoscopic surgery may provide a deeper insight into this condition. Of the seven cases, six occurred on the left side [5–7, 9–11], suggesting a certain tendency. Of the seven cases, bilateral ovarian prolapse was diagnosed preoperatively by ultrasonography in only two cases [5, 9]. We failed to diagnose the patient preoperatively. Computed tomography for pediatric inguinal hernias should be avoided in principle, and the examination is typically performed using ultrasonography only, which can make preoperative diagnosis difficult. These cases can present as potential challenges during surgical repair. Therefore, the possibility of bilateral ovarian herniation should be considered during hernia surgery in female infants.
